# Substrate utilisation of cultured skeletal muscle cells in patients with CFS

**DOI:** 10.1038/s41598-020-75406-w

**Published:** 2020-10-26

**Authors:** Cara Tomas, Joanna L. Elson, Julia L. Newton, Mark Walker

**Affiliations:** 1 Translational and Clinical Research Institute, Newcastle University, Newcastle upon Tyne, UK; 2 Centre for Human Metabolomics, North-West University, Potchefstroom, South Africa; 3 Newcastle upon Tyne Hospitals, NHS Foundation Trust, Newcastle upon Tyne, UK

**Keywords:** Mechanisms of disease, Biochemistry

## Abstract

Chronic fatigue syndrome (CFS) patients often suffer from severe muscle pain and an inability to exercise due to muscle fatigue. It has previously been shown that CFS skeletal muscle cells have lower levels of ATP and have AMP-activated protein kinase dysfunction. This study outlines experiments looking at the utilisation of different substrates by skeletal muscle cells from CFS patients (n = 9) and healthy controls (n = 11) using extracellular flux analysis. Results show that CFS skeletal muscle cells are unable to utilise glucose to the same extent as healthy control cells. CFS skeletal muscle cells were shown to oxidise galactose and fatty acids normally, indicating that the bioenergetic dysfunction lies upstream of the TCA cycle. The dysfunction in glucose oxidation is similar to what has previously been shown in blood cells from CFS patients. The consistency of cellular bioenergetic dysfunction in different cell types supports the hypothesis that CFS is a systemic disease. The retention of bioenergetic defects in cultured cells indicates that there is a genetic or epigenetic component to the disease. This is the first study to use cells derived from skeletal muscle biopsies in CFS patients and healthy controls to look at cellular bioenergetic function in whole cells.

## Introduction

Chronic fatigue syndrome (CFS), commonly known as Myalgic Encephalomyelitis (ME), is an unexplained illness with fatigue as the underlying symptom. The unexplained fatigue experienced by patients is not alleviated by rest or sleep and lasts for a minimum period of 6 months^1^. There are a myriad of other symptoms also associated with the disease. These include but are not limited to post-exertional malaise, myalgia, skeletal muscle fatigue, arthralgia, sleep disturbances, and cognitive impairment (‘brain fog’)^2^. The exact combination of symptoms that must be present differs between diagnostic criteria, with a universally accepted diagnostic criteria yet to be agreed upon^3^. Cellular bioenergetics have been studied previously in CFS^4–10^. These studies have used different cell types to show CFS patient cells have dysfunction of cellular energy production. Our group has previously used peripheral blood mononuclear cells (PBMCs) to show that CFS patient cells in the presence of glucose do not utilise the oxidative phosphorylation pathway (OXPHOS), also known as mitochondrial respiration, to the same extent as healthy controls^4^. This study also showed that not only was the impairment present at baseline levels, but CFS patient cells also had significantly lower levels of OXPHOS when the cells were pushed to their maximum using a cellular stressor. A subsequent study looked at the activity of individual mitochondrial respiratory chain complexes in both PBMCs and skeletal muscle cells and found all of them to be normal^5^.

Brown et al., used skeletal muscle cells derived from biopsies from CFS patients and age/sex matched healthy controls to show that unlike in healthy control cells, CFS cells do not activate the energy sensor AMP-activated protein kinase (AMPK) in response to in vitro exercise^11^. A follow up study however, showed that pharmacological activation of AMPK was successful in CFS cells leading to the suggestion that the inability of in vitro exercise to activate AMPK is caused by a defect proximal to AMPK^12^. The study also showed CFS cells to have significantly lower levels of ATP than healthy controls, even when AMPK was pharmacologically activated.

The two major cellular energy production pathways are OXPHOS and glycolysis. These can be investigated simultaneously using the seahorse extracellular flux analyser^13,14^. Having previously shown that CFS PBMCs have impaired OXPHOS in response to glucose, this technique is used in this study to investigate the ability of CFS skeletal muscle cells, and age/sex matched healthy control cells, to utilise different fuel sources for cellular energy production. Glucose, galactose, and fatty acid utilisation were all investigated during the course of this study. This study is the first to use CFS skeletal muscle cells to look at cellular bioenergetic function in whole cells.

## Results

### Participant characteristics

When muscle biopsies were collected fatigue impact scale (FIS) and BMI for all CFS patients were recorded. Age and gender were also recorded for each participant in the study. Participant characteristics are shown in Table 1.

**Table 1:** Characteristics for CFS patients and healthy controls on the day of muscle biopsy.

	Control	CFS
Gender (F:M)	9:2	8:1
Age (years)	51 ± 9.4	47 ± 9.6
FIS*	–	101 ± 22.3
BMI	–	27 ± 2.3

*FIS ≥ 40 is indicative of excessive symptomatic fatigue. FIS ≥ 80 indicates severe, symptomatic fatigue^15^.

### Glycolysis

**Figure 1:**
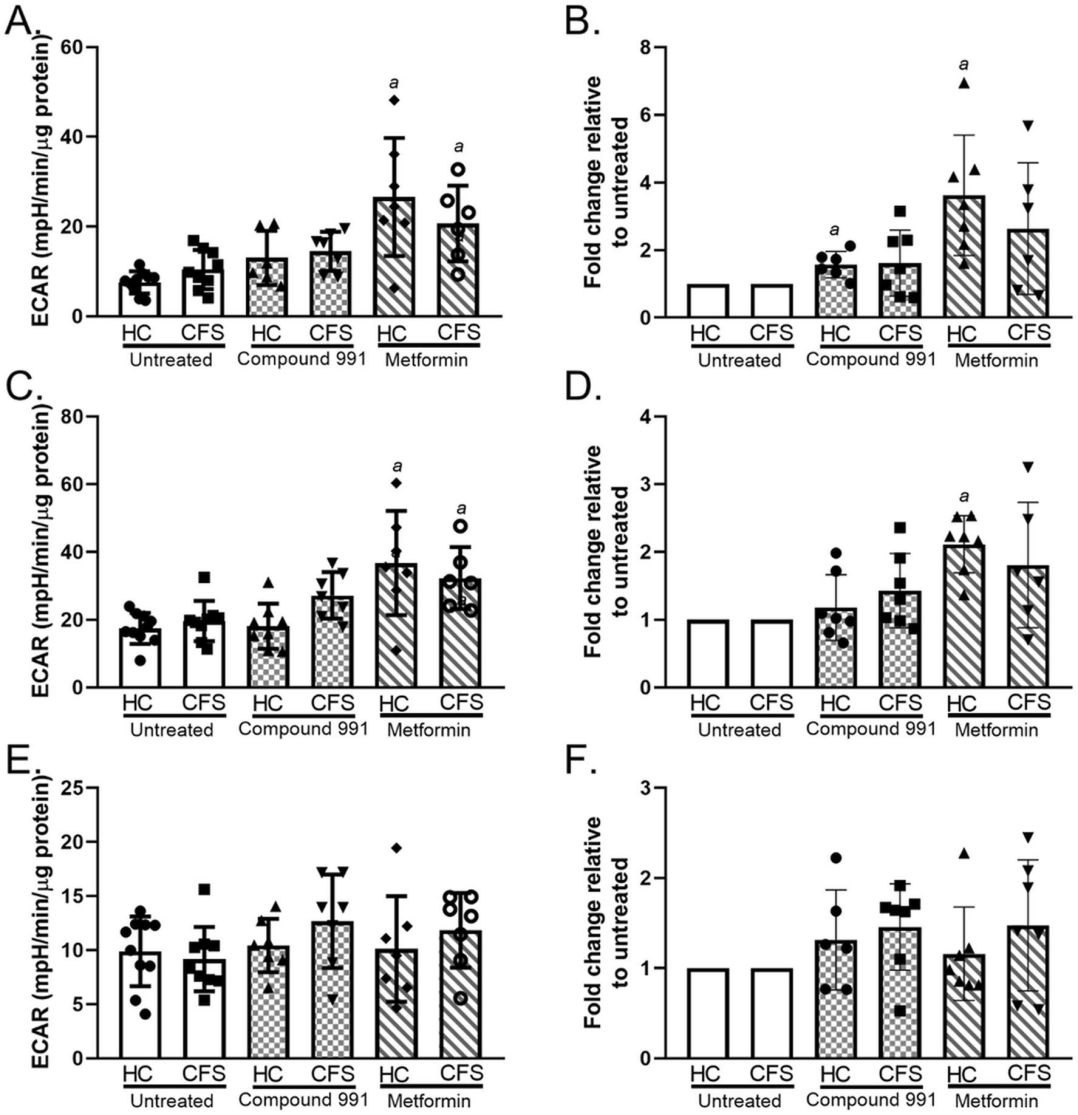
Results from a glycolytic stress test using CFS (n = 9) and healthy control (n = 10) skeletal muscle cells. Cells were untreated (white bars), treated with 1 µM compound 991 for 2 h (hatched bars), or treated with 2 mM metformin for 16 h (diagonal bars). For treated cells n = 7. (**A**) Glycolysis. (**B**) Glycolysis relative changes. (**C**) Glycolytic capacity. (**D**) Glycolytic capacity relative changes (**E**) Glycolytic reserve. (**F**) Glycolytic reserve relative changes. Groups were compared using two-way ANOVAs with post-hoc Bonferroni correction. *HC* healthy control; *CFS* CFS patient; *ECAR* extracellular acidification rate. a = differs significantly from untreated.

Glycolysis was assessed using extracellular flux analysis to obtain measures of glycolysis, glycolytic capacity, and glycolytic reserve for CFS and healthy control skeletal muscle cells. Results show there to be no significant differences between untreated CFS and control muscle cell utilisation of the glycolysis pathway (*p* ≥ 0.372) (Fig. 1). Cells were also treated with compound 991 (a direct AMPK activator) or metformin (an indirect AMPK activator) and glycolysis measured. Glycolysis, glycolytic capacity, and glycolytic reserve were shown to be comparable between the two groups even after compound 991 (*p* ≥ 0.129) or metformin (*p* ≥ 0.136) treatment. Metformin increased glycolysis and the glycolytic capacity of both cohort samples when compared to the untreated cells (*p* ≤ 0.008) (Fig. 1A,C). Both metformin and compound 991 treatment showed a trend towards a relative increase in glycolytic parameters in both cohorts (Fig. 1B,D,F), however, this was only statistically significant for healthy control glycolysis and glycolytic capacity (*p* ≤ 0.036).

### OXPHOS

#### Glucose

**Figure 2:**
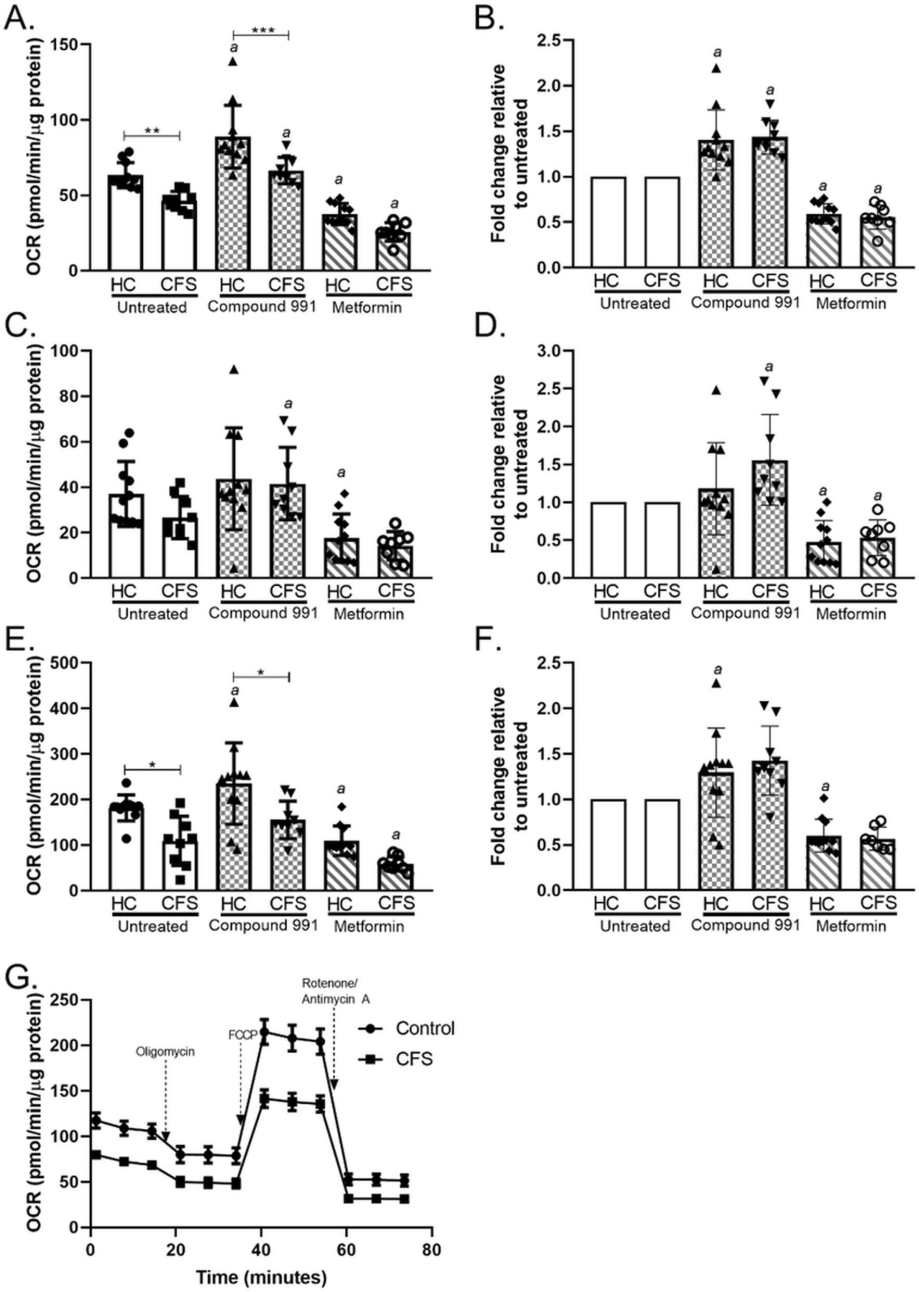
Mitochondrial stress test conducted using glucose (10 mM) as a substrate in skeletal muscle cells from CFS patients (n = 9) and healthy controls (n = 11). Cells were untreated (white bars), treated with 1 µM compound 991 for 2 h (hatched bars), or treated with 2 mM metformin for 16 h (diagonal bars). (**A**) Basal respiration. (**B**) Basal respiration relative changes. (**C**) ATP production. (**D**) ATP production relative changes. (**E**) Maximal respiration. (**F**) Maximal respiration relative changes. (**G**) Whole mitochondrial stress test trace in untreated cells. *OCR* oxygen consumption rate; *HC* healthy control; *CFS* CFS patient. Relative changes were calculated for each individual samples and compared to their untreated reading for each parameter. Groups were compared using two-way ANOVAs followed by post-hoc Bonferroni correction. *p < 0.05; **p < 0.01; ***p < 0.0001. a = differs significantly from untreated.

Using glucose as the primary substrate, OXPHOS was measured in untreated, compound 991 treated, and metformin treated CFS and healthy control skeletal muscle cells. Untreated CFS cells showed a significant reduction in the level of OXPHOS at baseline (*p* = 0.001) and when maximally stimulated (*p* = 0.002) to consume oxygen with glucose as a substrate (Fig. 2A,C). When cells were treated with metformin both healthy control and CFS muscle cells showed a reduction in OXPHOS parameters as expected with complex I inhibition. With compound 991 treatment the CFS cohort still had significantly lower levels of basal respiration (*p* < 0.001) and maximal respiration (*p* = 0.002) than healthy control cells. With metformin treatment the CFS cohort showed lower levels of basal respiration (*p* = 0.058) and maximal respiration (*p* = 0.074) but these results were not statistically significantly. There were no differences between CFS and healthy control groups in terms of ATP production. The treatment of cells with the direct AMPK activator compound 991 increased the basal respiration of CFS muscle cells to be comparable to that of untreated controls. This shows that compound 991 has the ability to restore OXPHOS function back to normal in isolated muscle cells. CFS cells were also comparable to healthy controls in terms of ATP production and maximal respiration when treated with compound 991. Metformin causes a comparable relative decrease in basal respiration between control and CFS cohorts. Equally, compound 991 caused comparable percentage increases in the two groups in terms of basal respiration showing that both cohorts are affected similarly by the two drugs.

#### Galactose

**Figure 3:**
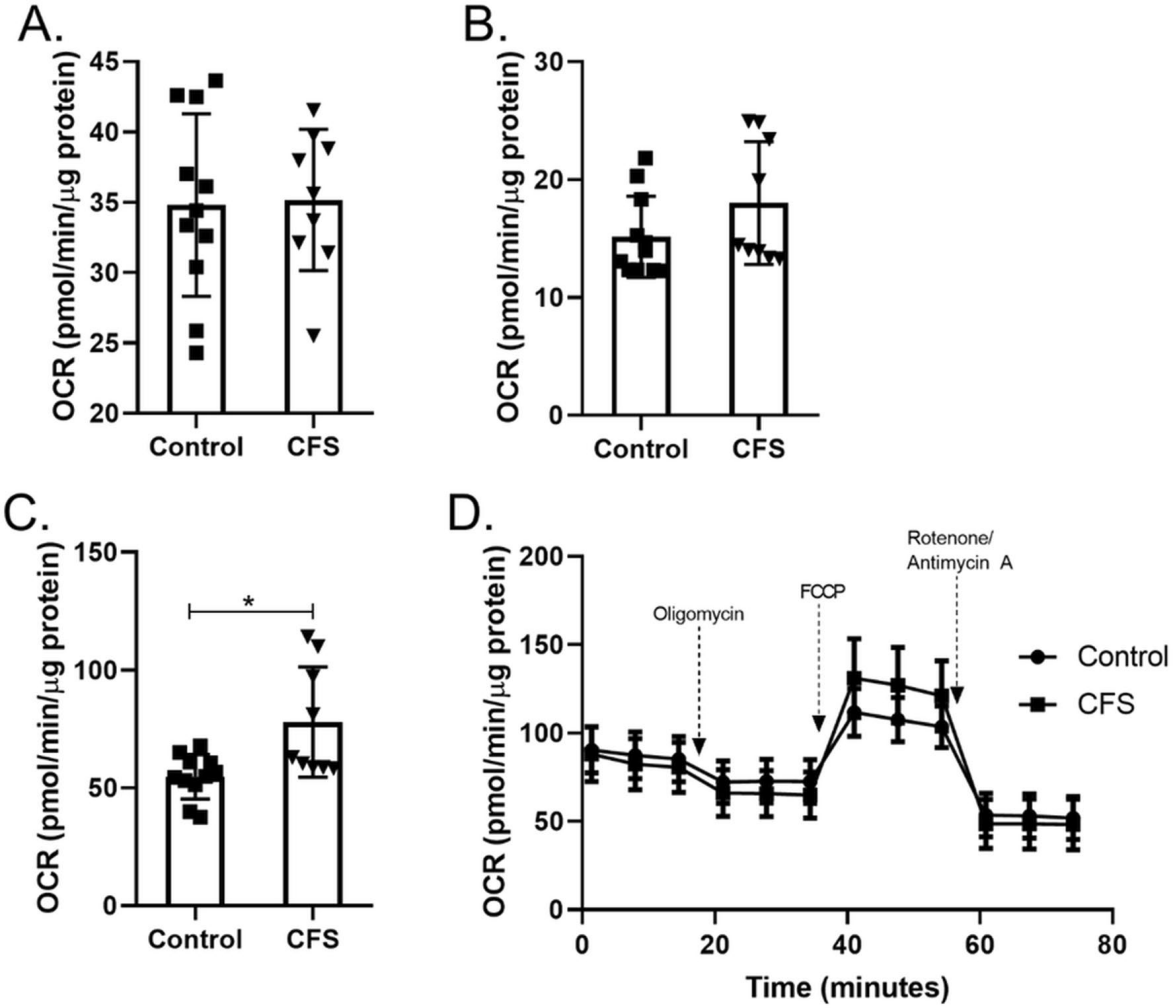
Mitochondrial stress test conducted using galactose (10 mM) as a substrate in skeletal muscle cells from CFS patients (n = 9) and healthy controls (n = 11). (**A**) Basal respiration). (**B**) ATP production. (**C**) Maximal respiration. (**D**) Whole mitochondrial stress test trace. OCR oxygen consumption rate. CFS and healthy control result*s were compared using Welch’s t* tests and adjusted for multiple comparisons. *p < 0.05.

The effect of using galactose as the primary substrate for the skeletal muscle cells was also investigated. CFS and healthy control cells were treated for 24 h with 10 mM galactose prior to OXPHOS being measured. Galactose forces cells to utilise OXPHOS to a greater extent than glucose, as the use of galactose does not result in a net gain of ATP via the glycolysis pathway thus forcing cells to use mitochondrial respiration in order to produce ATP^16^. Figure 3 shows that when using galactose as a substrate CFS skeletal muscle cells had normal levels of basal respiration and ATP production (*p* ≥ 0.180). This demonstrates that OXPHOS can function normally in these cells, but this is dependent on the substrate. CFS skeletal muscle cells incubated in galactose showed higher levels of maximal respiration than healthy control cells (*p* = 0.019). This increase indicates that under cellular energetic strain cells from a subset of CFS patients are able to increase their respiratory capacity depending on the substrates available.

#### Fatty acid oxidation

**Figure 4:**
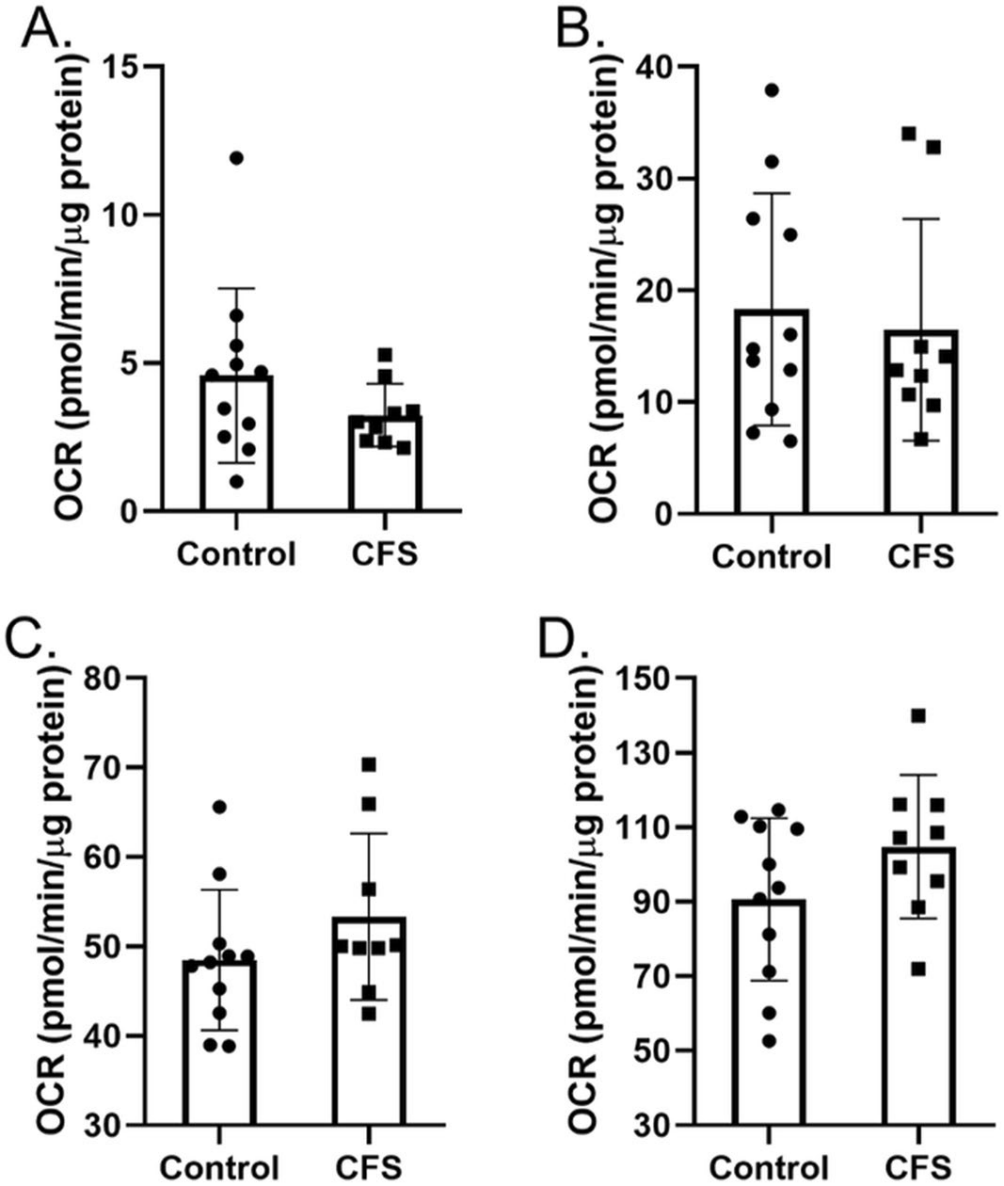
Oxidation of endogenous and exogenous fatty acids (palmitate) in skeletal muscle cells from CFS patients (n = 9) and healthy controls (n = 11). (**A**) Basal endogenous fatty acid oxidation. (**B**) Maximal endogenous fatty acid oxidation. (**C**) Basal exogenous fatty acid oxidation. (**D**) Maximal exogenous fatty acid oxidation. OCR oxygen consumption rate. Cohort*s* were compared using Welch’s *t* tests.

The ability of skeletal muscle cells to utilise fatty acids as a fuel source was also investigated. Beta oxidation of fatty acids does not require the glycolysis pathway as the acetyl-CoA produced enters the TCA cycle directly^17^. As we have shown that OXPHOS is dysfunctional in CFS skeletal muscle cells we wanted to narrow down the potential pathways included in the dysfunction. As the acetyl-coA produced from fatty acid oxidation enters the TCA cycle directly, this experiment allows us to look at whether the TCA cycle, and the processes downstream, are functioning normally. We calculated basal and maximal mitochondrial respiration rates for both endogenous and exogenous fatty acids. Figure 4 shows that there were no significant differences between the rates of fatty acid oxidation in control and CFS skeletal muscle cells (*p* ≥ 0.142).

### ROS

**Figure 5:**
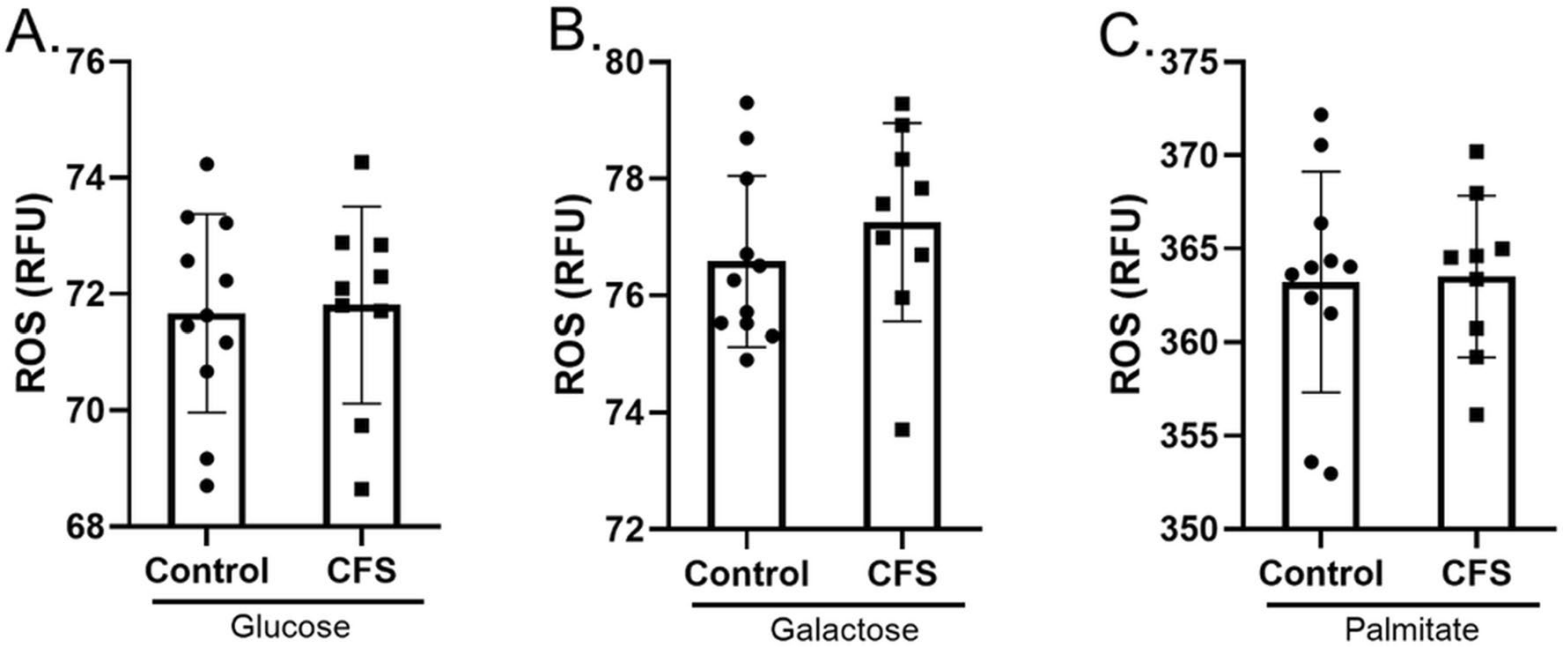
ROS production in skeletal muscle cells from CFS patients (n = 9) and healthy controls (n = 11) incubated for 24 h in (**A**) glucose. (**B**) Galactose. (**C**) Palmitate. Groups were compared using Welch’s *t* tests.

The production of ROS can significantly alter cellular oxygen consumption rates^18^. Oxygen consumption rate is the measurement used to calculate OXPHOS using extracellular flux analysis. Therefore, in order to show that the results from OXPHOS experiments shown in Figs. 1, 2, 3, 4 are due to mitochondrial respiration rather than ROS production, we measured ROS using the ROS detector DCFDA. ROS production was measured in the presence of glucose, galactose and palmitate:BSA (Fig. 5). In all three substrates we showed that there were no significant differences between the production of ROS from CFS skeletal muscle cells and healthy control skeletal muscle cells (*p* ≥ 0.390).

As there was no difference in ROS production between the two cohorts, this implies that the oxygen consumption recorded by the seahorse is not due to differences in ROS production and is therefore likely to be solely due to mitochondrial respiration.

## Discussion

This study has shown that the ability of CFS skeletal muscle cells to utilise glucose as a substrate is diminished compared to healthy control cells. However, CFS skeletal muscle cells were shown to oxidise galactose and fatty acids normally as well as demonstrating normal glycolytic function. The study indicates that the cellular bioenergetic dysfunction in CFS lies upstream of the TCA cycle.

We have shown normal functioning of the glycolysis pathway similar to earlier observations in PBMCs^4^. This shows a commonality between the two cell types taken from individuals with CFS.

This work has shown that CFS skeletal muscle cells have decreased OXPHOS compared to healthy control cells when glucose is used as a substrate. This is also similar to what has been shown previously in PBMCs^4^. When using glucose as a cellular substrate we treated skeletal muscle cells with two AMPK activators—compound 991 and metformin. Compound 991 (which is yet to be tested in clinical trials in humans) is a direct AMPK activator^12^. Compound 991 treatment appears to bring the OXPHOS levels of CFS patient cells up to the same level as untreated control cells. It is unclear whether this effect would extend to cells undergoing exercise in vitro using electrical pulse stimulation (EPS) as there is currently no way to incorporate EPS within seahorse experiments.

When comparing metformin treated cells to untreated cells, results showed CFS skeletal muscle cells were able to adapt to the lower energy production from mitochondria by increasing glycolytic functioning (Figs. 1, 2). This pattern is also seen in healthy control cells. The results in CFS cells shows that the cells have the capacity to increase their glycolytic function in response to decreased mitochondrial function, however, it is not clear why they cells are unable to do so when mitochondrial function is lower naturally rather than the reduced OXPHOS being caused by pharmacological intervention. It may be that metformin treatment causes OXPHOS to decrease to a threshold low enough to trigger the cellular response of increased glycolytic function to compensate. The ability of the CFS cells to increase glycolytic function in response to metformin treatment implies that AMPK is working as expected under resting conditions in CFS. Even though metformin treatment can increase glycolytic function in both control and CFS cohorts, the relative increase appears lower in the CFS cohort although the difference is not statistically significant. This still suggests a problem with the cells adaptive capacity, or inability to adapt as quickly, to changes in nutrient availability and energy production.

**Figure 6:**
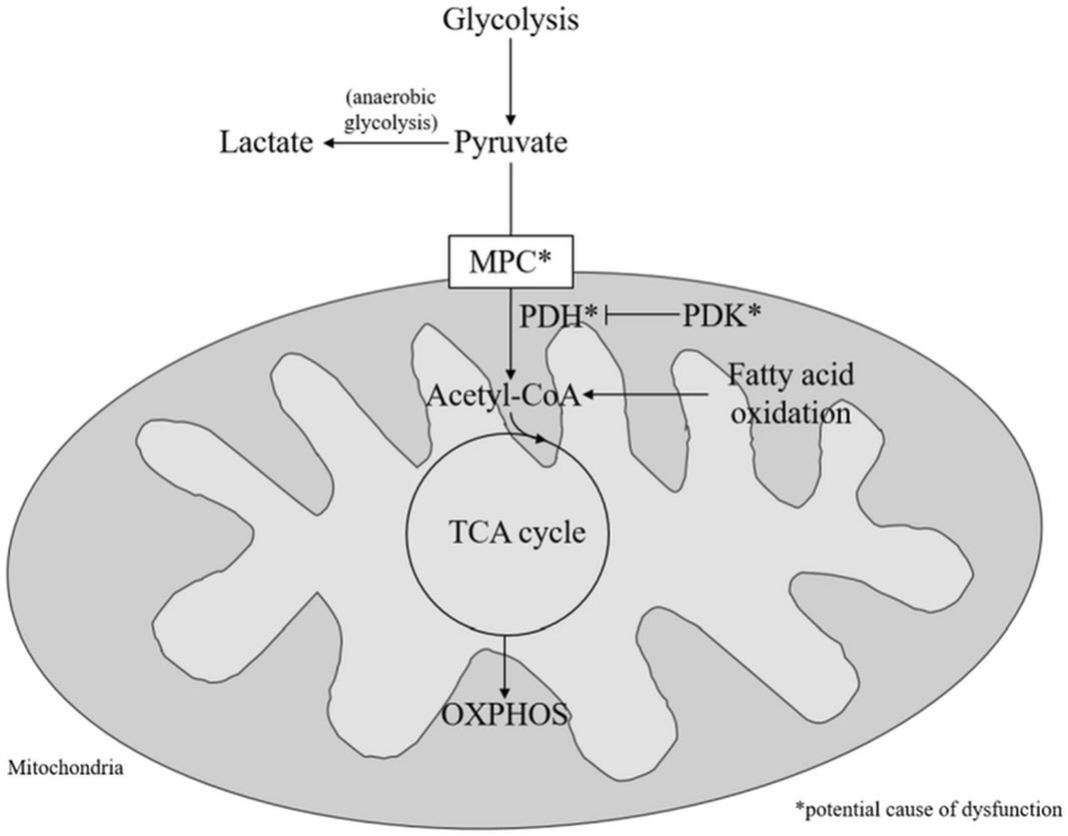
Figure showing potential molecular sites of dysfunction of glucose utilisation in CFS cells. MPC mitochondrial pyruvate carrier; PDH pyruvate dehydrogenase; PDK pyruvate dehydrogenase kinase; TCA tricarboxylic acid cycle; OXPHOS *oxidative phosphorylation*.

Oxidation of galactose provides no net increase of ATP from the glycolysis pathway, it is only when the substrate is utilised by the mitochondria during OXPHOS that ATP is produced^16^. This forces the cells to use OXPHOS alone for ATP production rather than also relying on the glycolysis pathway. Normal basal OXPHOS levels when using galactose as a substrate indicates normal functioning of the processes downstream of glycolysis such as the TCA cycle and mitochondrial respiratory complex function. This research supports previously published data showing normal functioning of individual mitochondrial respiratory chain complexes and work that found no proven pathogenic mitochondrial DNA variations in CFS and implies that any defect is upstream of the mitochondrial respiratory complexes themselves^5,19^. The ability of some of the CFS patient cells to increase maximal respiration above that of healthy controls when galactose is used as a substrate shows a stratification effect within the patient population. This may indicate that some patients are able to adapt to changing substrate availability better than others. However, it should be noted that in the galactose experiments there was no exogenous glucose provided in the cell medium. This would have limited the UDP-glucose substrate pool which is required for the galactose uridyl transferase reaction and the conversion of galactose to G-6-P. Different results may be seen with the addition of exogenous glucose to the cell medium. Fatty acid oxidation was also normal in the CFS skeletal muscle cells, which again implies that any defect is upstream of the TCA cycle as beta-oxidation produces acetyl-CoA which can enter the TCA cycle directly. The normal basal functioning of CFS skeletal muscle cells when using galactose or fatty acids as substrates, coupled with normal glycolytic functioning may indicate that the problem is in the link step between pyruvate and the TCA cycle. This could potentially include defects to pyruvate dehydrogenase complex (PDH), pyruvate dehydrogenase kinase (PDK), or mitochondrial protein carriers (Fig. 6). This fits with a study which used a PDK inhibitor in an open-label proof-of-principle trial and a study by *Fluge *et al. looking at PDH dysregulation in CFS blood samples^7,20^. The in vitro study by *Fluge *et al. used amino acid and mRNA analysis which suggested that mitochondrial pyruvate oxidation may be abnormal in CFS^7^. Using galactose as a cellular substrate shows an increase in maximal respiration and reserve capacity in CFS skeletal muscle cells compare to healthy controls. This increase was also seen by *Fluge *et al. when then treated control muscle cells with CFS patient serum. They postulated that it may be due to a PDH deficiency.

A study by van Loon et al. looking at muscle fuel utilisation has shown that at rest oxidation of fatty acids provides 56% of energy and carbohydrates provide 44%^21^. This changes slightly to 55% and 46% respectively at 40% of the maximal workload for each individual. For higher intensity exercise (75% of maximal workload) the ratio switches again to be 24% of energy provided by fatty acid oxidation and 76% from carbohydrate oxidation. The propensity of muscle to utilise carbohydrates to a greater extent during exercise coupled with the inability of CFS patient cells to utilise glucose oxidation to the same extent as healthy controls may explain, at least in part, why CFS patients suffer from post-exertional malaise.

Experiments in this study were designed to investigate the oxidation and utilisation of individual substrates one by one. However, this does not completely reflect the reality of substrates present in patients. For example, given the deficiencies in the ability of CFS cells to utilise glucose as a substrate we would expect to see a shift towards beta oxidation of fatty acids in order to compensate for the deficiencies^22^. However, these experiments show that does not appear to be happening. It may be that deficiencies in CFS mitochondrial function are due to an inability to switch between substrate oxidation as effectively or quickly as healthy control cells.

This study suggests that AMPK is functioning normally at rest as the cells are able to match control OXPHOS when given galactose as a fuel source. Additionally, CFS skeletal muscle cells are able to adapt to inhibition of mitochondrial complex I by metformin by increasing glycolysis accordingly. This does not contradict the work published by *Brown *et al. which showed that exercise cannot properly activate skeletal muscle AMPK ^11^. But rather, this work indicates there may be two separate issues in the energy production processes within CFS patient cells—the activation of AMPK in response to exercise, and the ability of cells to utilise OXPHOS in response with glucose as a substrate at rest. Even for moderately intense exercise, glucose accounts for about 50% of the fuel utilised for ATP production^21^. This, along with the inability of AMPK to become activated by exercise, may be why patients are unable to exercise properly—as they are both unable to produce energy properly from glucose and the AMPK is not telling the cells there is not enough energy. These two factors will leave patients muscle cells with a depletion of ATP during exercise.

This study, using skeletal muscle cells, also building on work our group has previously done looking at cellular bioenergetic function in CFS using PBMCs^4^. There are many similarities between the two cell types in terms of the way in which patient cells utilise bioenergetic pathways. Both cell types showed comparable rates of glycolysis (when looking at the CFS cohort as a whole). Additionally, both PBMCs and skeletal muscle cells from CFS patients had significantly lower mitochondrial functioning when utilising glucose as a fuel source. Lower ATP production from mitochondria was also observed in both cell types^4,12^. The similar dysfunction in bioenergetic functioning between the two cell types indicates that CFS is a multi-system disease. This is in line with patient’s experience of the disease^23^. The PBMCs analysed previously and the skeletal muscle cells used in these experiments were not from the same patients or controls. Moving forward it would be helpful to collect blood samples and muscle biopsies from participants on the same day in order to look at the correlations between PBMC and skeletal muscle cell bioenergetics in individual patients. Additionally, samples collected longitudinally should also be looked at.

This is the first study to use cells derived from skeletal muscle biopsies in CFS patients and healthy controls to look at cellular bioenergetic function in whole cells. The use of patient derived skeletal muscle cells is vital for furthering our understanding of how the disease affects patients given that many suffer from muscle pain and fatigue as a core component of their illness^24^. This work illustrates the importance of skeletal muscle cells cultured from patient and control biopsies are a vital resource in uncovering the biochemical causes of the disease.

## Conclusion

CFS skeletal muscle cells show similar dysfunction in mitochondrial respiration as PBMCs. The inability of CFS cells to utilise glucose as a fuel source to the same extent as healthy controls results in decreased mitochondrial respiration at both basal and maximal levels. The ability of CFS skeletal muscle cells to utilise galactose and fatty acids to the same extent as healthy controls suggests that the dysfunction is in the link step between glycolysis and the TCA cycle. The similarities between dysfunction seen previously in PBMC and those seen here in muscle cells suggests that CFS is a multi-tissue disease, which reflects what patients report and the symptoms they present with.

## Methods

All methods were carried out in accordance with relevant guidelines and regulations.

### Study subjects

Muscle biopsies obtained from the *vastus lateralis* were collected from 9 CFS patients and 11 age/sex matched controls. On the day of biopsy, CFS patients completed a fatigue impact scale (FIS) questionnaire so that functional limitations due to fatigue could be measured^25^. Age, gender and body mass index (BMI) of patients were also recorded. For healthy control participants only age and gender information was collected. Participants were recruited via the Newcastle NHS clinic for CFS at the Newcastle Hospitals NHS foundation Trust. All CFS patients fulfilled the Fukuda diagnostic criteria^1^. All participants provided written informed consent. Ethical approval was granted by the Newcastle and North Tyneside Joint Ethics Committee.

### Reagents

Oligomycin (Sigma Aldrich), FCCP (Sigma Aldrich), Rotenone (Sigma Aldrich), Antimycin A (Sigma Aldrich), Extracellular flux microplates (Agilent Technologies), Extracellular flux cartridges (Agilent Technologies), DCFDA (Sigma Aldrich), Bradford reagent (Sigma Aldrich), Lysis buffer (Sigma Aldrich), Ham’s F10 medium (Fisher Scientific 11574436), chick embryo extract (Life Science Production), amphotericin B (Sigma Aldrich), penicillin–streptomycin (Sigma Aldrich), minimum essential medium (Sigma Aldrich M2279), Glucose-free minimum essential medium (SLS LZBE12-611F), glucose (Sigma Aldrich), galactose (Sigma Aldrich), 2-deoxy-glucose (Sigma Aldrich), Palmitate conjugated to BSA (Agilent technologies), BSA (Agilent technologies), Etomoxir (Sigma Aldrich), PBS (Sigma Aldrich), compound 991 (donated from AstraZeneca), metformin (Sigma Aldrich).

### Cell culture

Muscle biopsies from the vastus lateralis were obtained from the participants, and muscle precursor cells isolated as described previously^26^. Myoblasts were cultured up to passage 7 in Ham’s F10 medium supplemented with 20% (v/v) FBS, 2% (v/v) chick embryo extract, 1% (v/v) penicillin/streptomycin and 1% (v/v) amphotericin B. When cells reached confluence at passage 7 media was changed to induce differentiation into myotubes. Differentiation media was comprised of minimum essential media supplemented with 2% (v/v) FBS, 1% (v/v) penicillin/streptomycin and 1% (v/v) Amphotericin B. Differentiation was confirmed by observing cell fusion using a light microscope. All experiments were performed after 7 days of differentiation.

### Extracellular flux analysis

Extracellular flux analysis assessing glycolysis and OXPHOS was performed using the XFe96 by Agilent Technologies according to manufacturer’s protocols and as described in Tomas et al.^4^. For each protocol three basal measurements were made with an additional three measurements taken after the addition of each compound. Each compound was made up to a 10 × concentration. Each measurement consists of a mix/wait/measure process of 3/0/3 min respectively. All experiments were performed at 37 °C.

For glycolysis and OXPHOS experiments myoblast cells were seeded on a specialist microplate (Agilent Technologies) at 30,000 cells/well and differentiated for 7 days. Each sample was seeded in at least quadruplicate. On the day of experiments cells were given fresh media in a volume of 180 µl. The final volume after the addition of all of the compounds was 247 µl (180 µl media, 20 µl of first compound added, 22 µl of second compound added, 25 µl of third compound added).

To determine glycolysis extracellular acidification rate (ECAR) was recorded following the sequential addition of 10 mM glucose, 1 µM oligomycin, and 50 mM 2-deoxy-glucose. Measurements of glycolysis were adjusted for non-glycolytic acidification using the following equation determined by Mookerjee et al.^27^:

$$ {\text{PPR}}_{{{\text{glyc}}}} = {\text{ECAR}}_{{{\text{tot}}}} {\text{/BP}} - \left( {{1}0^{{({\text{pH}} - {\text{pK1}})}} /\left( {{1 + 1}0^{{({\text{pH}} - {\text{pK1}})}} } \right)} \right)\left( {{\text{max H}}^{ + } {\text{/O}}_{{2}} } \right)\left( {{\text{OCR}}_{{{\text{tot}}}} - {\text{OCR}}_{{{\text{rot}}/{\text{myx}}}} } \right) $$

Data was analysed according to manufacturer’s instructions allowing glycolysis (average of three measurements post-glucose addition − average of three basal measurements), glycolytic capacity (average of three measurements post oligomycin addition − average of basal measurements), and glycolytic reserve (glycolytic capacity − glycolysis) to be determined^4^.

To determine OXPHOS, oxygen consumption rate (OCR) of cells was recorded following the sequential addition of 2 µM oligomycin, 2 µM Carbonyl cyanide-4-(trifluoromethoxy)phenylhydrazone (FCCP), and 0.5 µM rotenone/antimycin A. Data was analysed according to manufacturer’s instructions allowing basal respiration (average of three basal measurements − average of three measurements post rotenone/antimycin A treatment), ATP production (average of basal measurements − average of three measurements post oligomycin addition), and maximal respiration (average of three measurements post FCCP − average of three measurements post rotenone/antimycin A addition) to be determined^4^.

For OXPHOS experiments using glucose or galactose, cells were pre-treated with either 10 mM glucose or 10 mM galactose for 24 h before experiments were performed. The media used for this 24 h period was MEM without glucose which was supplemented with glucose or galactose. For experiments using compound 991, cells were treated with 1 µM compound 991 for 2 h prior to the experiment. For experiments using metformin as a treatment, cells were treated with 2 mM metformin for 16 h prior to performing the experiment. These times and concentrations were previously used by Brown et al.^11,12^.

For OXPHOS experiments investigating fatty acid oxidation (FAO) were performed according to manufacturer’s instructions with the exception of the 24 h substrate restriction. Briefly, 45mins prior to the assay cells were washed twice with FAO assay medium (111 mM NaCl, 4.7 mM KCl, 1.25 mM CaCl_2_, 2 mM MgSO_4_, 1.2 mM NaH_2_PO_4_, 2.5 mM glucose, 0.5 mM carnitine, 5 mM HEPES). FAO assay medium was added to cells and the plate incubated in a non-CO_2_ incubator for 45miutes. This time allows the metabolically active myotubes to deplete any exogenous FA that may exist within the media so that only endogenous FA remain.15mins prior to the start of the assay 40 µM of etomoxir (eto) or FAO medium were added to the appropriate wells and the plate incubated in a non-CO_2_ incubator for 15mins. Just prior to starting the assay Palmitate-BSA (final in-well concentrations of 200 µM palmitate conjugated to 33.3 µM BSA) or BSA (final in-well concentration of 33.3 µM) were added to the appropriate wells. Palmitate-BSA was used to look at exogenous FA utilisation, and BSA was used to look at endogenous FA utilisation. The addition of etomoxir was used to inhibit CPT1 so that FA oxidation cannot take place. The experiment was run with sequential addition of 2.5 µM oligomycin, 2 µM FCCP, and 0.5 µM rotenone/antimycin A as described above. Data was analysed according to manufacturer’s instructions using Wave software (version 2.6).

All data were normalised to total protein concentration.

### Reactive oxygen species detection

Reactive oxygen species (ROS) were measured using 2′,7′–Dichlorofluorescin diacetate (DCFDA). After 7 days of differentiation, media was removed and cells washed with PBS. Substrates were added (10 mM glucose, 10 mM galactose, or 100 µM palmitate conjugated to 17 µM BSA) and the plate incubated for 24 h (for palmitate assay was run immediately). 20 µM DCFDA was added to cells and incubated at 37 °C, 5% CO_2_ in the dark for 45 min. DCFDA was removed and cells washed with PBS. PBS supplemented with 10% FBS was added to cells and the plate incubated for 30 min at 37 °C and 5% CO_2_. Fluorescence was measured (excitation 485 nm; emission 535 nm) using a Molecular Devices SpectraMax M5R and SoftMax Pro 7.0 software. Each measurement was performed in triplicate. Data were normalised to total protein concentration.

### Normalisation to protein concentration

Data from all experiments were normalised for total protein concentration using a Bradford assay. Medium was removed from cells and 25 µl of ice-cold lysis buffer added. Cell detachment was ensured by scraping and checked under a microscope. 10 µl of sample/standard were added to a new 96-well plate in triplicate. 200 µl of Bradford reagent was added to each well, including standards. The plate was placed on a plate shaker for 15 min at room temperature. Absorbance was read at 595 nm. Protein concentration was determined using the standard curve created from the standards.

### Statistical analysis

All results are shown as mean ± SD. Data was analysed using two-way ANOVAs or Welch’s *t* tests where appropriate. Data was tested for equal variances using Levene’s test. All *p* values were corrected using post-hoc Bonferroni correction for multiple comparisons. *p* < 0.05 was considered significant. Statistical analysis was performed using GraphPad Prism software (version 8).

## Data Availability

The datasets used in this study are available from the corresponding author on reasonable request.
